# Genetic and Phenotypic Variations Within Ancient ‘Mehras’ Olive (*Olea europaea* L.)

**DOI:** 10.3390/ijms27115087

**Published:** 2026-06-04

**Authors:** Ruba M. Al-Mohusaien, Monther T. Sadder, Ebrahem Al-Taha’at, Bandar N. Hamadneh, Orowah A. Al-Slaibi, Hamad A. Alkhatatbeh, Farah Abu Siam

**Affiliations:** 1 Department of Plant Production, Smart and Sustainable Agriculture, Faculty of Agriculture, Ajloun National University, Ajloun 26810, Jordan; r.al-mohusien@anu.edu.jo; 2 Department of Horticulture and Crop Science, School of Agriculture, University of Jordan, Amman 11942, Jordan; 3 Department of Agricultural Extension and Marketing, Faculty of Agriculture, Ajloun National University, Ajloun 26810, Jordan; e.altahat@anu.edu.jo (E.A.-T.); o.slaibi@anu.edu.jo (O.A.A.-S.); 4 Department of Human Nutrition and Dietetics, Faculty of Agriculture, Ajloun National University, Ajloun 26810, Jordan; ba.hamadneh@anu.edu.jo; 5 Ash-Shoubak University College, Al-Balqa Applied University, Al-Salt 19117, Jordan; h.khatatbeh@bau.edu.jo; 6 Department of Agricultural Biotechnology and Genetic Engineering, Faculty of Agricultural Technology, Al-Ahliyya Amman University, Amman 19111, Jordan; f.abusiam@ammanu.edu.jo

**Keywords:** *Olea europaea*, ‘Mehras’, olive, DNA markers, genetic diversity, phenotypic traits, agricultural heritage

## Abstract

Ancient olives are considered a major resource of gene pool, adapted across ages to ever changing environments. The ancient ‘Mehras’ olive is the only cultivar inscribed on the UNESCO Representative List of the Intangible Cultural Heritage Of Humanity as recognized for its longevity and historical significance. However, detailed genetic and phenotypic analyses are still missing. Nineteen ‘Mehras’ accessions were collected from northern Jordan and subjected to inter-simple sequence repeat (ISSR) marker analysis and to a set of phenotypic parameters (leaf, fruit, and stone). ISSR analysis revealed similarity values ranging between 0.53 and 1.00, indicating moderate to high genetic diversity. Bayesian clustering and dendrogram analyses identified two major genetic clusters with limited admixture, indicating extended clonal propagation in addition to the transport of planting material. Phenotypic parameters revealed tangible variation among accessions, with major influence of fruit and stone traits followed by leaf traits. Strong correlations were observed between key traits, including fruit width and flesh thickness, while an inverse relationship was observed between flesh and stone percentages. Multivariate analysis further revealed clear separation among investigated accessions. The Mantel test showed a moderate correlation between genetic and phenotypic distances. Spatial analysis suggested weak geographic structuring of diversity, indicating exchange of plant material. ‘Mehras’ olive harbors structured genetic and phenotypic diversity influenced mainly by adaptation and traditional cultivation practices. These findings provide a foundation for conservation, breeding, and sustainable utilization of this ancient and culturally significant cultivar.

## 1. Introduction

Ancient olive trees (*Olea europaea* L.) are one of the most vital fruit trees in the Mediterranean and represent invaluable genetic resources for the future [1]. They are characterized by longevity and tolerance to drought and salinity; therefore, they could survive for centuries under diverse climatic conditions [1,2]. In addition, ancient olives have strongly influenced Mediterranean landscapes, agricultural traditions, and rural livelihoods for millennia [3,4,5]. Olive oil derived from these trees is a key component of the Mediterranean diet and is recognized for its high content of monounsaturated fatty acids and phenolic compounds associated with significant health-promoting properties [6]. Ancient olive trees also possess considerable cultural value through their association with traditional farming practices, culinary heritage, and agro-tourism activities [7,8].

The ancient ‘Mehras’ cultivar, also known as ‘Al-Mihrass’, is one of the oldest recognized olive cultivars in Jordan and is notable for its longevity, resilience, and distinctive morphological characteristics such as elongated leaves and medium-sized fruits [9,10]. Traditional knowledge associated with grafting, pruning, harvesting, and maintaining these trees has been transmitted across generations and recently contributed to the inscription of the ‘Al-Mihrass’ tree on the UNESCO Representative List of the Intangible Cultural Heritage of Humanity [11]. Despite this cultural and historical importance, the cultivar remains insufficiently characterized at the genetic and phenotypic levels.

The eastern Mediterranean and Levant regions are considered primary centers of olive domestication and diversification, from which cultivated olives spread westward across the Mediterranean Basin, resulting in numerous locally adapted genotypes [1,12]. Although modern olive production is currently dominated by high-yielding commercial cultivars, ancient olive populations preserved in traditional orchards and marginal environments continue to represent invaluable genetic resources [3,13,14,15]. These ancient genotypes have undergone long-term selection under stressful environmental conditions and therefore harbor unique adaptive traits associated with drought tolerance and resilience [10]. Consequently, sustainable conservation of ancient olive germplasm has become increasingly central for future breeding and conservation [16,17].

Recent studies have demonstrated that ancient olive cultivars frequently possess unique genetic structures and distinctive phenotypic traits [1,10,16,18]. Intensive characterization efforts combining molecular markers and morphological analyses have therefore become essential tools for understanding diversity within ancient olive germplasm [1,19,20,21]. Several Mediterranean studies have emphasized ancient olive trees and evaluated their genetic distinctiveness [20,21]. However, in Jordan, recent surveys of ancient olives were limited to relatively few locations with intensive within-site sampling, resulting in the identification of only a limited number of unique haplotypes [1]. As a result, the extent of genetic and phenotypic variation within the ancient ‘Mehras’ cultivar remains poorly understood.

The present study addresses this gap through an integrated molecular and phenotypic characterization of historic ‘Mehras’ olive accessions collected from northern Jordan. We hypothesized that: (i) ancient ‘Mehras’ accessions retain substantial genetic and phenotypic diversity despite long-term clonal propagation; (ii) molecular and morphological variation exhibit measurable correspondence; and (iii) geographic proximity does not necessarily predict genetic similarity due to historical human-mediated movement of planting material. The findings of this study are expected to support future conservation, germplasm management, and utilization strategies for this historically vital olive cultivar.

## 2. Results

### 2.1. Genetic Analysis

ISSR marker analysis revealed differences between the ancient ‘Mehras’ olive accessions (Supplementary Figure S1). Pairwise similarity coefficients ranged from 0.53 to 1.00, indicating the presence of both genetically distinct accessions and nearly identical genotypes within the population. The average similarity value was 0.77, suggesting moderate to high genetic diversity despite the mainly vegetative mode of propagation. Some accession pairs showed high similarity (1.00), which likely reflects clonal propagation. These accessions are clustered in one clade in the dendrogram.

On the other hand, the lowest similarity value (0.53) was observed between accessions 8 and 12. In fact, the accessions 1, 8, 11 and 12 showed lower similarity values with most other accessions. And hence, they are genetically distant from the remaining accessions. The majority of pairwise similarities were between 0.65 and 0.85, reflecting gradual genetic variation rather than distinct genetic separation among accessions. Polymorphic relationships represented a moderate portion of total variation, a clear indicator of the presence of genetic diversity between ancient ‘Mehras’ olives.

**Figure 1:**
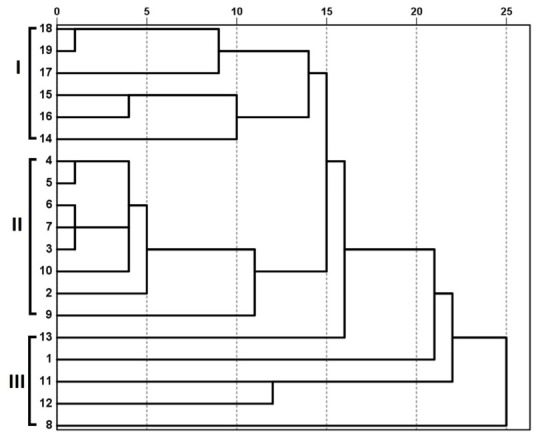
Dendrogram for ‘Mehras’ accessions collected from northern parts of Jordan.

The UPGMA dendrogram grouped the investigated ‘Mehras’ olive accessions from northern Jordan into three main clusters (I–III) (Figure 1). Cluster I grouped six accessions, which were genetically related but were separated from other accessions in clusters II and III.

Cluster II contained the largest number of accessions (eight) and included some closely related accessions forming clear sub-clusters with short branch lengths, indicating a relatively higher genetic similarity within this sub-cluster. Finally, cluster III grouped just five olive accessions and had relatively longer branch distances, reflecting limited genetic similarity. Among all investigated ‘Mehras’ olive accessions, accession 8 was the most distant one, forming a separate branch, indicating lower similarity with the remaining accessions. The dendrogram revealed clear genetic structuring among the studied accessions. It is important to notice that the reference ‘Mehras’ accession 19 was grouped in cluster I, leading to selecting the remaining clusters II and III for more divergent accession, of different genetic makeup, possibly nominating with new names.

**Figure 2:**
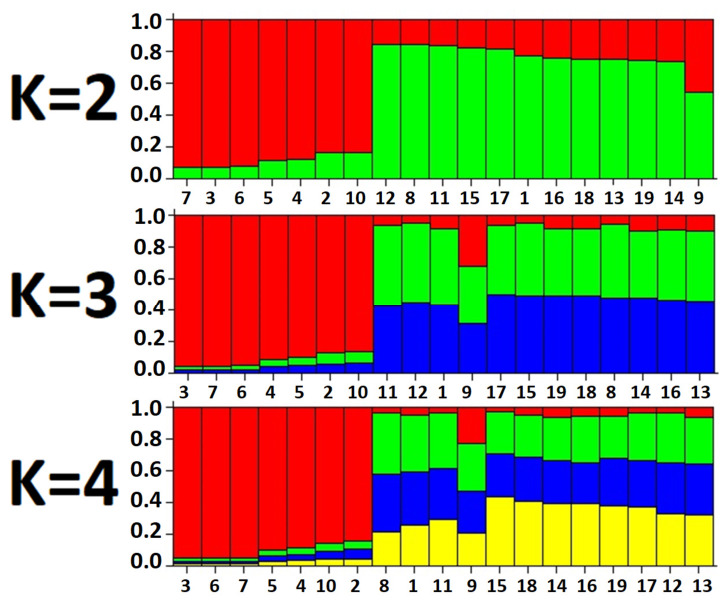
Structure analysis for ‘Mehras’ accessions K range (2–4).

Bayesian clustering analysis using STRUCTURE revealed a clear subdivision of the 19 investigated ‘Mehras’ olive accessions into two major genetic groups (K = 2) with limited admixture (Figure 2). The most likely number of clusters, determined using the Evanno ΔK method, showed a clear peak at K = 2, confirming this subdivision as the optimal population structure. Increasing the number of clusters (K = 3 or K = 4) resulted in progressive fragmentation of one major group without distinct genetic boundaries, indicating over-clustering rather than a biologically meaningful differentiation. In addition, the UPGMA dendrogram (Figure 2) supported the presence of two major clusters (I and II), which is in agreement with the STRUCTURE results, whereas the third cluster (III) with long branches reflects variation rather than actual separate grouping.

DNA ISSR markers revealed variable levels of polymorphism and genetic diversity for the investigated ‘Mehras’ olive accessions (Table 1). Polymorphism information content (*PIC*) values ranged from 0.37 (ISSR_842) to 0.78 (ISSR_840), with moderate to high values between them. Similarly, the discrimination power (*D*) varied between 0.39 and 0.82, with ISSR_840 exhibiting the highest ability to differentiate olive accessions. Observed heterozygosity (*Ho*) ranged from 0.38 to 0.72, whereas expected heterozygosity (*He*) values were much lower, ranging from 0.11 to 0.23. Shannon’s diversity index (*H′*) showed moderate diversity across loci, with values between 0.26 and 0.45. Among the investigated DNA markers, ISSR_840 and ISSR_834 showed higher polymorphism and diversity parameters, while ISSR_842 showed the lowest values.

### 2.2. Phenotypic Parameters

The leaf, fruit, and stone phenotypic traits were averaged for five trees and listed with SD in Supplementary Table S1 for all 19 ancient olive accessions.

**Figure 3:**
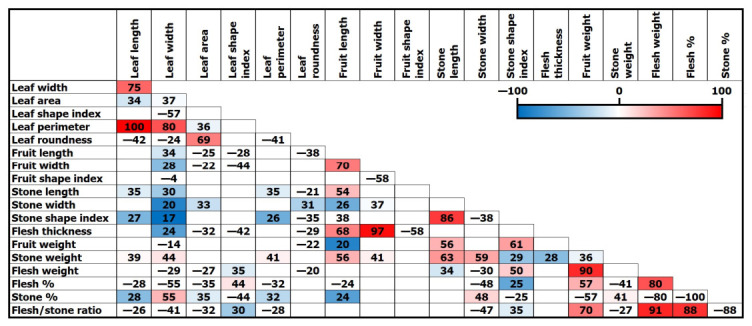
Pearson correlation heatmap among leaf, fruit, and stone phenotypic traits of ancient ‘Mehras’ olive accessions. Significance values were adjusted using the Benjamini–Hochberg false discovery rate (FDR) correction method. Only correlations remaining significant after correction (adjusted *p* < 0.05) are shown.

Pearson correlation analysis revealed significant and biologically meaningful relationships among leaf, fruit, and stone parameters (Figure 3). Following Benjamini–Hochberg false discovery rate (FDR) correction across all 171 pairwise comparisons, 101 correlations initially identified as significant at *p* < 0.05 remained significant after adjustment, indicating that the detected relationships were robust and unlikely to represent false-positive associations.

Strong correlations were observed among vegetative traits, with leaf length showing a positive correlation with leaf perimeter (r = 1.00) and a strong correlation with leaf width (r = 0.75). Leaf roundness was negatively correlated with leaf length (r = −0.42) and leaf perimeter (r = −0.41), indicating that elongated leaves tended to exhibit lower roundness values. Fruit-related parameters exhibited unique correlations. Fruit width showed a very strong positive correlation with flesh thickness (r = 0.97) and a strong correlation with fruit length (r = 0.68). Fruit weight was strongly correlated with flesh weight (r = 0.90) and flesh percentage (r = 0.80), reflecting the role of flesh development in determining fruit mass. Stone-related traits displayed inverse relationships with flesh traits. Flesh percentage was negatively correlated with stone percentage (r = −1.00), indicating a biased allocation of assimilated toward flesh rather than stone tissues. The flesh-to-stone ratio showed strong positive correlations with flesh percentage (r = 0.91) and fruit weight (r = 0.70), highlighting its relevance as an integrative indicator of fruit quality and productivity. However, weaker correlations should be interpreted cautiously because some associations may still reflect environmental variation or statistical noise associated with high-dimensional trait analysis.

**Figure 4:**
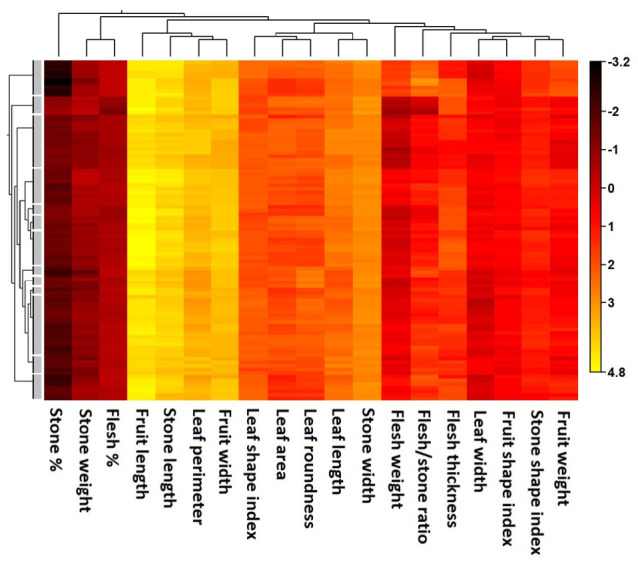
Hierarchical clustering heatmap of historic ‘Mehras’ olive accessions based on standardized phenotypic traits.

Hierarchical clustering of the ancient ‘Mehras’ olive accessions based on quantified phenotypic parameters revealed clear phenotypic structuring (Figure 4). The heatmap and dendrogram grouped the accessions into several clusters, reflecting similarities in fruit, stone, and leaf parameters. Traits related to fruit and stone size, including fruit weight, flesh weight, stone weight and flesh/stone ratio, contributed strongly to cluster discrimination and showed contrasting patterns across clusters. In addition, leaf phenotypic parameters (leaf length, width, area, roundness, and shape index) displayed small variability and supported sub-clustering. Clustering of traits highlighted either increases and decreases in trait values among clusters, indicating the presence of both large-fruited (high-flesh) accessions and smaller-fruited (stone-dominant) phenotypes within the investigated ancient ‘Mehras’ olive accessions.

The relationship between genetic and phenotypic variation was assessed using a Mantel test comparing pairwise ISSR-based genetic distances with phenotypic distance matrices (Supplementary Figure S2). The Mantel test revealed a weak but statistically significant positive association between the two datasets (r = 0.312, *p* = 0.021; 9999 permutations). The scatterplot showed a general increase in phenotypic distance with increasing genetic distance, although substantial dispersion among pairwise comparisons was observed. These results indicate that ISSR-based genetic divergence explains only a limited proportion of the observed phenotypic variation among the evaluated ‘Mehras’ olive accessions.

**Figure 5:**
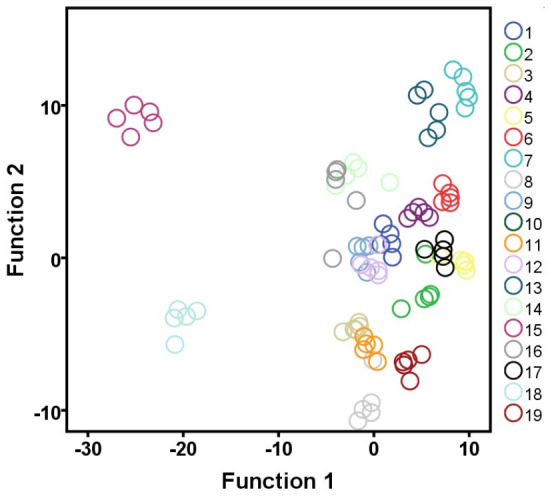
Canonical discriminant functions between olive ‘Mehras’ accessions (1–19) from cluster analysis and the two main functions were performed on the basis of 19 phenotypic parameters.

Discriminant function analysis (DFA) based on quantified phenotypic parameters revealed clear multivariate separation among the ancient ‘Mehras’ olive accessions. (Figure 5). The first two discriminant functions could discriminate among investigated accessions, with Function 1 accounting for the majority of the variation and providing the strongest separation along the horizontal axis. Several accessions formed well-defined and compact clusters, indicating high within-group similarity, whereas others showed partial overlap in the central region of the plot. Distinct groups located at the extremes of the ordination space demonstrated marked phenotypic divergence, reflecting substantial differences in fruit, stone, and leaf characteristics. Overall, the DFA scatterplot illustrates structured phenotypic diversity and provides clear discrimination among most of the investigated accessions.

Spatial interpretation of genetic and phenotypic data revealed a mixed distribution of ‘Mehras’ olive accessions across northern Jordan (Supplementary Figure S3). Genetically and phenotypically similar accessions were not consistently geographically clustered, while neighboring accessions exhibited higher similarities. The weak correspondence between geographic proximity and genetic or phenotypic similarity indicates limited spatial structuring of diversity and suggests that geographic distance alone does not explain observed variation among ancient ‘Mehras’ olives.

## 3. Discussion

This study provides a comprehensive assessment of genetic and phenotypic diversity within accessions of the ancient ‘Mehras’ olive cultivar in northern Jordan. Unlike earlier surveys that focused on limited geographic regions with intensive within-site sampling [1], the current investigation integrated molecular and phenotypic analyses across all available accessions, providing broader insight into intra-cultivar diversity and population structure. Despite the predominantly clonal mode of propagation traditionally associated with olive cultivation, the investigated accessions exhibited considerable genetic and phenotypic variability. Similar levels of intra-cultivar variation have been reported in ancient Mediterranean olive germplasm, where long-term vegetative propagation combined with somatic mutation, local adaptation, and human-mediated selection contributed to detectable divergence among accessions [22,23,24,25]. The results therefore support the hypothesis that ancient olive cultivars represent dynamic and heterogeneous genetic resources rather than genetically uniform clonal lineages.

### 3.1. Genetic Analysis

The observed clustering patterns revealed high genetic similarity among several accessions within the ‘Mehras’ cultivar, while simultaneously identifying genetically distinct lineages. In particular in clusters II and III, as they did not include the ‘Mehras’ reference accession number 19 as in cluster I. The predominance of accessions within cluster II likely reflects a shared genetic background resulting from long-term clonal propagation and the exchange of planting material among nearby regions, a phenomenon widely documented in cultivated olives across the Mediterranean Basin [22]. Similar clustering structures were previously reported in Greek, Italian, and Albanian olive germplasm, where traditional propagation practices contributed to high within-cultivar similarity despite substantial regional diversity [23,26]. In contrast, the distinct separation of cluster III, characterized by longer branch lengths, suggests the presence of divergent accessions that may have originated from ancestral lineages or accumulated mutations during centuries of vegetative propagation [23]. Ancient olive trees are known to accumulate somatic mutations over long lifespans, which can generate detectable genetic divergence even within historically recognized cultivars [27].

The unique divergence of accessions 1, 8, 11, and 12 further supports the existence of rare or genetically differentiated lineages within the ‘Mehras’ germplasm. Similar observations have been reported in surveys of ancient Jordanian olives, where a limited number of genetically distinct haplotypes contributed disproportionately to overall population structure [1]. Such divergent accessions represent remnants of ancient propagation events, localized adaptation processes, or introgression from neighboring olive populations. Comparable findings were also documented in Mediterranean olive collections where rare genotypes preserved unique allelic combinations and contributed significantly to germplasm diversity [22,23,24].

The combined STRUCTURE, Evanno ΔK, and UPGMA analyses consistently supported the existence of two principal genetic groups, whereas higher K values generated only weak sub-structuring attributable to allele-frequency differences or admixture rather than discrete evolutionary populations. Similar patterns of weak differentiation and mixed ancestry have frequently been observed in ancient olive populations due to centuries of germplasm exchange and movement of propagative material among regions [24]. Olive domestication and subsequent spread throughout the Mediterranean involved repeated hybridization and introgression events between cultivated and wild forms, producing complex population structures characterized by partial admixture rather than strict genetic isolation [24]. Therefore, the observed population structure of ‘Mehras’ olives appears consistent with the broader evolutionary history of Mediterranean olive cultivation.

The relatively high polymorphism information content (PIC) and discrimination power values obtained for the ISSR markers demonstrate their suitability for diversity assessment and cultivar discrimination in ancient olive germplasm. Similar studies on Mediterranean olives have shown that ISSR markers provide reliable detection of polymorphism and remain useful for preliminary germplasm characterization, especially in clonally propagated crops [25]. Although SSR and SNP markers generally provide higher genomic resolution, ISSR markers remain cost-effective and informative for assessing intracultivar variation and identifying genetically distinct accessions [22,25]. The moderate Shannon diversity and heterozygosity estimates observed in this study are consistent with expectations for clonally propagated perennial crops, where diversity mainly originates from somatic mutation and occasional sexual recombination rather than extensive meiotic reshuffling [26,27]. Comparable diversity levels have been reported in other ancient olive populations from Mediterranean countries, supporting the concept that traditional olive germplasm retains detectable but moderate genetic variability despite long-term vegetative propagation [22,23,24].

Nevertheless, the relatively limited number of ISSR primers used in the present study should be considered when interpreting the genetic structure results. Although the selected primers generated clear, reproducible, and polymorphic banding patterns with sufficient discriminatory power to distinguish the investigated accessions, ISSR markers represent anonymous dominant loci and provide only partial genome coverage. Consequently, the detected population structure and diversity estimates should be regarded as preliminary assessments of intra-cultivar variation. Higher-resolution marker systems such as SSRs, SNP arrays, or genome-wide sequencing approaches would likely provide finer-scale resolution of genetic relationships, admixture patterns, and adaptive variation within ancient ‘Mehras’ olive germplasm. Similar limitations associated with reduced marker numbers have also been acknowledged in previous olive diversity studies employing ISSR markers [14,23].

### 3.2. Phenotypic Parameters

The Mantel analysis revealed a weak but statistically significant positive correlation between molecular and phenotypic distances (r = 0.312), indicating that ISSR-based genetic divergence only partially reflects phenotypic differentiation among the investigated ‘Mehras’ accessions. The relatively low explanatory power of this relationship suggests that phenotypic variability is strongly influenced by additional factors beyond neutral molecular polymorphisms, including environmental conditions, genotype–environment interactions, developmental plasticity, and quantitative inheritance of morphological traits [28]. Similar weak genotype–phenotype relationships have been reported in other clonally propagated perennial crops and olive germplasm collections, where substantial phenotypic variation may arise despite relatively limited genome-wide divergence [29]. Therefore, the observed association should be interpreted cautiously as evidence of partial rather than strong correspondence between molecular and phenotypic datasets. These findings further emphasize the importance of integrating molecular and phenotypic analyses to obtain a comprehensive understanding of olive diversity and cultivar structure [30].

It should also be considered that ISSR markers are largely anonymous neutral markers and are not necessarily linked to genomic regions controlling adaptive or quantitative phenotypic traits. Consequently, ISSR-based genetic distances may only partially reflect functional genetic variation underlying morphological differences among accessions. Therefore, the relatively weak Mantel correlation observed in the present study is biologically reasonable and should not be interpreted as a direct measure of genotype-phenotype causality.

The hierarchical clustering analysis demonstrated substantial phenotypic diversity within the ‘Mehras’ accessions, with fruit and stone characteristics representing the major determinants of clustering patterns. Similar findings have been reported in Mediterranean olive germplasm studies, where fruit weight, stone morphology, flesh percentage, and fruit dimensions were among the most informative descriptors for cultivar discrimination [28,30]. In the present study, accessions exhibiting higher flesh weight and flesh-to-stone ratios formed distinct phenotypic groups, suggesting potential agronomic relevance for oil production and fruit quality. Leaf morphological variability further indicated adaptive or developmental divergence among accessions, potentially reflecting local environmental conditions and long-term selection pressures [29]. Such phenotypic differentiation is characteristic of ancient farmer-maintained germplasm, where centuries of cultivation under diverse ecological conditions contribute to the emergence of locally adapted morphotypes [1,29].

The observed phenotypic diversity is quantitatively comparable to previous studies of ancient olive cultivars from Sicily, Greece, and other Mediterranean regions, where multivariate analyses consistently revealed detectable intracultivar divergence despite common cultivar designation [23,28]. Caruso et al. [28], for example, demonstrated considerable clonal variation among Sicilian olives based on both morphological traits and molecular markers, supporting the interpretation that traditional olive cultivars are dynamic populations rather than genetically fixed entities. Similarly, studies on ancient Mediterranean olive trees reported substantial variability in fruit size, stone characteristics, and leaf morphology associated with local adaptation and environmental heterogeneity [22,23,24]. Therefore, the phenotypic variability detected in the current study likely reflects a combination of genetic divergence, somatic mutation accumulation, and environmental influence acting over long cultivation histories.

Discriminant function analysis (DFA) further confirmed the strong discriminatory value of phenotypic traits for differentiating among ancient ‘Mehras’ accessions. The formation of compact and relatively well-separated clusters indicates phenotypic consistency within certain accessions, whereas broader dispersion among groups reflects divergence accumulated through somatic mutation, local adaptation, and historical farmer selection [29]. Partial overlap among clusters likely reflects shared ancestry or convergence of traits under similar environmental conditions. Comparable DFA and multivariate clustering results have been reported in other olive germplasm characterization studies, where combined morphological descriptors significantly improved cultivar discrimination compared with single-trait approaches [28,30]. The integration of multiple correlated phenotypic variables therefore provides a robust framework for identifying and characterizing ancient olive germplasm.

### 3.3. Historical Context and Gene Flow

The observed genetic structuring likely reflects centuries of traditional olive cultivation and germplasm movement throughout northern Jordan. Clonal propagation preserves core cultivar identity, whereas occasional sexual reproduction, somatic mutation, and human-mediated exchange of propagative material introduce additional diversity over time [22,24]. Similar patterns of low-to-moderate diversity combined with partial admixture have been documented in numerous ancient Mediterranean olive populations shaped by centuries of cultivation and regional trade [22,23,24]. The existence of genetically divergent accessions within the ‘Mehras’ cultivar underscores the complex evolutionary history of ancient olive trees, where environmental adaptation, farmer selection, and localized propagation practices collectively shaped present-day diversity [1,23]. The weak relationship between geographic proximity and genetic similarity further supports the important role of human-mediated dispersal in distributing olive propagules across the region independently of spatial distance.

### 3.4. Conservation and Breeding Implications

The combined molecular and phenotypic diversity identified within the ancient ‘Mehras’ olive germplasm highlights its considerable value for conservation and breeding programs. Ancient olive accessions harbor unique alleles associated with tolerance to abiotic stresses, adaptation to marginal environments, disease resistance, and desirable fruit quality traits [25,26]. Such traits are increasingly becoming essential under current climate change scenarios and the growing need for resilient agricultural systems. Similar conclusions have been reached in studies of Mediterranean ancient olives, where heritage germplasm was recognized as an invaluable reservoir of adaptive diversity for future cultivar improvement [22,23,24].

Preservation of these ancient accessions is therefore essential not only for maintaining cultural and historical heritage but also for safeguarding valuable genetic resources for future breeding efforts. Integrating molecular markers with detailed phenotypic characterization enables more informed conservation strategies, including both in situ preservation of historic orchards and ex situ germplasm maintenance. The present study further supports the importance of continued characterization of ancient Jordanian olive germplasm to facilitate sustainable utilization, cultivar authentication, and long-term conservation of this historically and agriculturally significant genetic resource.

## 4. Materials and Methods

### 4.1. Plant Material

**Figure 6:**
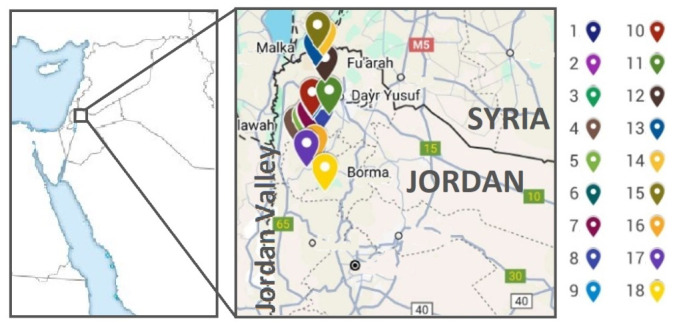
Geographic distribution of ancient ‘Mehras’ olive locations across northern Jordan.

This study was conducted using historic ‘Mehras’ olive tree (*Olea europaea* L.) accession cultivated in traditional olive-growing regions of northern Jordan. A total of nineteen ‘Mehras’ accessions (including the reference sample number 19 [1]) were selected based on their historical recognition, long-term cultivation, and maintenance under traditional management systems (Figure 6). Accession information is presented in Table 2. The sampling sites covered a latitudinal range (32.21–32.72° N) and a longitudinal range (35.66–35.80° E), including both spatially clustered and geographically dispersed locations.

For each accession, five representative trees were selected to capture within-location variability while maintaining balanced sampling among sites. Trees were chosen based on health status, apparent age, typical ‘Mehras’ morphological characteristics, and absence of severe pruning or disease symptoms. Young and healthy leaves were collected from each tree individually for molecular analysis, while fruits were harvested at physiological maturity for phenotypic characterization. Molecular analyses were initially conducted on individual trees and subsequently integrated at the accession level, where identical DNA patterns were found in all samples from each accession; therefore, one profile was used per accession. On the other hand, phenotypic measurements were averaged across the five sampled trees to obtain representative accession values. All samples were labeled according to accession number and sampling location to ensure traceability throughout the study.

### 4.2. DNA Extraction

Genomic DNA was extracted from fresh leaf tissue using a modified cetyltrimethylammonium bromide (CTAB) protocol optimized for olive leaves. Leaf tissue was ground in liquid nitrogen, of which 100 mg was used for DNA extraction. DNA concentration and purity were assessed spectrophotometrically, and DNA integrity was verified by 0.8% agarose gel electrophoresis.

### 4.3. DNA Amplification

Inter-simple sequence repeat (ISSR) amplification was performed using polymerase chain reaction (PCR). Four primers were selected for ISSR analysis (Table 3). ISSR primers (UBC series, University of British Columbia, Vancouver, BC, Canada) were used following the standard ISSR-PCR methodology described [31,32]. Each reaction was conducted in a final volume of 20 μL containing 10 μL of 2× PCR master mix (Trans-Gen Biotech, Beijing, China), 2 μL primer (10 μM), 2 μL template DNA (5 ng/μL), and 6 μL nuclease-free water, assembled in 0.2 mL PCR tubes. Amplifications were carried out in a Veriti thermal cycler (Applied Biosystems, Foster City, CA, USA) with an initial denaturation at 95 °C for 5 min, followed by 45 cycles of denaturation at 95 °C for 30 s, annealing at 50 °C for 45 s, and extension at 72 °C for 1 min, with a final extension at 72 °C for 10 min. PCR products were separated by electrophoresis on 1.8% agarose gels in 1× TBE buffer at 90 V for 150 min. Fragment sizes were estimated using a 100 bp DNA ladder (BIO-HELIX, New Taipei, Taiwan). Bands were stained with RedSafe™ nucleic acid stain and visualized under UV illumination using an Alpha Imager 2200 gel documentation system (Alpha Innotech, San Leandro, CA, USA). The annealing temperature of 50 °C was selected following preliminary optimization experiments to ensure clear and reproducible banding patterns across all ISSR primers used in this study. Since ISSR primers are generally anchored and differ in sequence composition, a uniform annealing temperature was adopted as a compromise to allow consistent amplification conditions across all reactions. This approach is widely applied in ISSR-based genetic diversity studies to maintain comparability among primers while ensuring reliable amplification of polymorphic loci.

The ISSR primers used in this study were selected following preliminary screening based on amplification reproducibility, band clarity, and polymorphism level. A total of 34 polymorphic bands were generated (Table 3), which provided sufficient discriminatory power to resolve genetic relationships among the investigated ‘Mehras’ accessions. Comparable numbers of ISSR primers have been successfully applied in previous olive diversity and cultivar characterization studies [31,32]. Higher-resolution marker systems such as SSRs and SNPs may provide additional insights into fine-scale population structure and evolutionary history.

### 4.4. Molecular Data Analysis

Only clear, bright, and reproducible ISSR bands were scored for analysis, and each amplification was repeated at least three times to ensure reproducibility. Bands were recorded in a binary matrix as present (1) and absent (0). A similarity matrix was calculated using Jaccard similarity coefficients [33] using SPSS v22 (IBM Corp., Armonk, NY, USA), and a dendrogram was constructed using the unweighted pair-group method with arithmetic mean (UPGMA).

Markers were assessed by calculating polymorphism information content (*PIC*) according to [34] using *PIC* = 2*f*(1 − *f*), where f represents the frequency of band presence. Discriminating power (*D*) of each primer was estimated following [35] to evaluate its ability to differentiate investigated accessions. Observed heterozygosity (*Ho*) was estimated as band frequency, while expected heterozygosity (*He*), equivalent to Nei’s gene diversity [36], was calculated assuming Hardy–Weinberg equilibrium as *He* = 2*p*(1 − *p*). Genetic diversity was further quantified using Shannon’s information index (*H′* = −∑[*p* ln *p* + *q* ln *q*]), where *p* and *q* represent the frequencies of band presence and absence, respectively [37].

Population structure was inferred using STRUCTURE v2.3.4 [38] under an admixture model with correlated allele frequencies. The number of genetic clusters (K) was evaluated from K = 2 to 4 without prior population information. For each K, analyses were run with a burn-in period of 30,000 iterations followed by 30,000 Markov Chain Monte Carlo (MCMC) replications [39].

### 4.5. Phenotypic Data

At harvest, fruits and leaves were collected from five representative trees per location. Leaf phenotypic traits measured included leaf length, leaf width, leaf area, leaf shape index (length/width), leaf perimeter calculated using Ramanujan’s equation [40], and leaf roundness estimated according to Russ’s formula [41]. Fruit traits included fruit length, fruit width, fruit shape index (length/width), and fruit weight. Stones were manually separated using an olive pitter, disinfected in a 5% solution for 15 min, thoroughly rinsed with water, and dried with paper towels prior to measurement. Stone traits recorded were stone length, stone width, stone shape index (length/width), stone weight, and stone percentage (stone weight/fruit weight). Flesh characteristics included flesh thickness, flesh weight, flesh percentage (flesh weight/fruit weight), and flesh-to-stone ratio (*w*/*w*).

All measured traits were used for subsequent statistical analyses. Canonical discriminant analysis (CDA) was conducted using SPSS v18. Hierarchical clustering based on Manhattan distance was performed to generate a heatmap illustrating phenotypic relationships among traits and accessions.

Because multiple pairwise correlations were calculated among the 19 phenotypic parameters (171 pairwise comparisons), significance values were adjusted using the Benjamini–Hochberg false discovery rate (FDR) correction method to reduce the probability of false-positive associations arising from multiple testing. Only correlations with adjusted *p*-values < 0.05 were considered statistically significant and retained for interpretation and visualization. Correlation analysis was primarily intended as an exploratory approach to identify major biological relationships among morphological traits.

### 4.6. Correlation Between Genetic and Phenotypic Data

Phenotypic and ISSR marker datasets were analyzed to find genetic-phenotypic relationships among the ancient olive accessions. Both datasets (quantitative phenotypic traits and ISSR binary matrix) were imported and processed in R v4.4.0 [42]. Statistical analyses and visualization were conducted using base R and the packages vegan [43], phangorn [44], dendextend [45], and ggplot2 [46]. Pairwise distance matrices were calculated using Euclidean distance for phenotypic variables and Jaccard distance for ISSR presence–absence data. Hierarchical clustering was performed using the unweighted pair-group method with arithmetic mean (UPGMA). The association between molecular and phenotypic distance matrices was evaluated using a Mantel test with 9999 permutations [47]. Principal component analysis (PCA) was additionally applied to the phenotypic dataset to visualize patterns of variation and grouping among accessions.

## 5. Conclusions

The integrative analysis of genetic and phenotypic variation in the investigated ancient ‘Mehras’ olive accessions demonstrates that, despite long-term clonal propagation, this cultivar harbors substantial diversity. ISSR markers revealed clear genetic structuring, with accessions 1, 8, 11, and 12 showing the highest genetic divergence and therefore representing priority candidates for conservation due to their distinct genetic backgrounds. Phenotypic analyses indicated that fruit- and stone-related traits, particularly fruit weight, flesh percentage, stone percentage, and flesh-to-stone ratio, provide the strongest discriminatory power among accessions and are therefore most informative for breeding and germplasm characterization. The moderate but significant correlation between molecular and phenotypic data highlights the combined influence of genetic background and environmental factors in shaping observable traits. Weak geographic structuring suggests that historical human-mediated exchange of planting material has played a major role in maintaining and redistributing diversity across northern Jordan. These findings underscore the value of ‘Mehras’ olives as a reservoir of genetically and phenotypically distinct germplasm, supporting targeted conservation of highly divergent accessions and the use of key fruit quality traits in future breeding programs. Although the present ISSR dataset provided sufficient resolution for preliminary diversity assessment, future studies using larger marker sets and high-resolution genomic approaches together with multi-environment phenotyping are recommended to refine genetic relationships, detect adaptive loci, and assess trait stability under different environmental conditions.

## Figures and Tables

**Table 1:** PIC, D, Ho, He and H′ for ISSR markers used in olive accessions.

Marker	*PIC*	*D*	*Ho*	*He*	*H′*
ISSR_840	0.78	0.82	0.52	0.23	0.44
ISSR_842	0.37	0.39	0.38	0.11	0.26
ISSR_855	0.73	0.77	0.58	0.17	0.36
ISSR_834	0.73	0.77	0.72	0.14	0.45

**Table 2:** Geographic coordinates and locations of the ancient ‘Mehras’ olive locations sampled in northern Jordan.

Acc.	Region	Latitude	Longitude
1	Ain Al-Bustan	32.303534	35.738541
2	Wadi Kufranjeh	32.303427	35.707508
3	Al-Maysar Al-Hashemiyah	32.365499	35.665914
4	Halawah	32.382696	35.659063
5	Wadi Al Rayan	32.399696	35.696556
6	Al-Kourah Judayta	32.416663	35.712662
7	Burqush	32.418161	35.717032
8	Zoubia	32.434861	35.769604
9	Der Abi Saeed	32.469800	35.728441
10	Tibnah	32.482104	35.726635
11	Dayr Yusuf	32.489434	35.800763
12	Fu’arah	32.604504	35.782769
13	Malka	32.658759	35.743691
14	Saham	32.697609	35.775603
15	Al-Yarmouk Battle Location	32.720912	35.750853
16	Wadi Al-Tawaheen	32.317315	35.738425
17	Kufranjeh	32.299387	35.701220
18	Borma	32.214147	35.782222
19	Al-Hashemiyah	32.365745	35.663258

**Table 3:** ISSR markers and sequence, number of polymorphic markers and unique bands (Y = C or T).

ISSR Marker	Sequence 5′–3′	Number of Polymorphic Bands
ISSR_840	(AG)8C	9
ISSR_842	(AG)8G	5
ISSR_855	(AG)8YT	8
ISSR_834	(AC)8YG	12
Total		34

## Data Availability

The original contributions presented in this study are included in the article/Supplementary Material. Further inquiries can be directed to the corresponding author.

## Supplementary Materials

The following supporting information can be downloaded at: https://www.mdpi.com/article/10.3390/ijms27115087/s1.

## Abbreviations

The following abbreviations are used in this manuscript:

ISSR	inter-simple sequence repeat
D	discrimination power
He	expected heterozygosity
Ho	Observed heterozygosity
H′	Shannon’s diversity index
PIC	Polymorphism information content
