# Supplementary material for: Genetic and Phenotypic Variations Within Ancient ‘Mehras’ Olive (Olea europaea L.)
Source: Int J Mol Sci. 2026 Jun 4;27(11):5087. doi: 10.3390/ijms27115087 (PMC13257465; doi:10.3390/ijms27115087)
Supplement: Supplementary file 1 [file ijms-27-05087-s001.zip › ijms-4283666-supplementary.pdf]

Supplementary Table S1. Phenotypic traits (leaf, fruit, and stone) of ancient 'Mehras' olive accessions, data represents means (n = 5) and SD underneath.

Accession	Leaf length	Leaf width	Leaf area	Leaf shape index	Leaf perimeter	Leaf roundness	Fruit length	Fruit width	Fruit shape index	Stone length	Stone width	Stone shape index	Flesh thickness	Fruit weight	Stone weight	Flesh weight	Flesh %	Stone %	Flesh/stone ratio
**1**	5.08	1.14	3.86	4.46	10.78	4.49	18.00	12.37	1.45	16.00	7.75	2.06	2.31	1.81	0.50	1.31	0.72	0.28	2.64
	0.13	0.05	0.52	0.13	0.29	0.51	1.87	0.09	0.14	1.00	0.19	0.09	0.05	0.16	0.05	0.16	0.03	0.03	0.43
**2**	5.22	1.26	3.31	4.18	11.16	3.72	24.00	12.71	1.89	18.00	6.79	2.65	2.96	1.64	0.49	1.15	0.70	0.30	2.37
	0.53	0.21	0.50	0.33	1.19	0.27	1.00	0.13	0.06	1.58	0.11	0.19	0.01	0.23	0.07	0.20	0.04	0.04	0.48
**3**	5.76	1.26	4.45	4.59	12.20	4.59	18.40	10.15	3.56	17.00	6.95	2.44	1.60	1.83	0.47	1.37	0.74	0.26	2.93
	0.09	0.09	0.26	0.24	0.25	0.21	1.52	4.87	4.56	1.58	0.19	0.17	2.38	0.24	0.04	0.23	0.03	0.03	0.52
**4**	4.28	0.90	3.57	4.78	9.03	4.97	20.00	12.69	1.58	16.20	7.59	2.13	2.55	1.74	0.54	1.20	0.69	0.31	2.27
	0.26	0.10	0.16	0.27	0.58	0.27	0.00	0.11	0.01	1.30	0.21	0.11	0.05	0.02	0.06	0.06	0.04	0.04	0.39
**5**	5.72	1.52	3.01	3.81	12.36	3.07	26.40	15.33	1.72	19.40	7.46	2.60	3.94	1.61	0.59	1.02	0.63	0.37	1.79
	0.18	0.23	0.15	0.43	0.53	0.24	1.14	0.08	0.07	1.52	0.34	0.10	0.13	0.13	0.10	0.16	0.07	0.07	0.55
**6**	5.06	1.46	4.27	3.49	11.04	4.87	25.00	15.51	1.61	16.80	7.41	2.26	4.05	1.54	0.58	0.96	0.61	0.39	1.80
	0.21	0.17	0.43	0.26	0.55	0.56	2.12	0.11	0.13	1.92	0.21	0.20	0.05	0.24	0.13	0.32	0.12	0.12	0.90
**7**	5.70	1.66	5.02	3.47	12.46	5.09	23.20	16.49	1.41	16.40	8.59	1.91	3.95	1.54	0.73	0.81	0.52	0.48	1.17
	0.25	0.22	0.56	0.31	0.69	0.65	0.84	0.11	0.04	1.14	0.23	0.08	0.06	0.17	0.10	0.24	0.10	0.10	0.50
**8**	6.94	1.62	4.35	4.31	14.79	3.68	17.80	12.39	1.44	15.00	7.07	2.12	2.66	1.50	0.48	1.02	0.68	0.32	2.16
	0.47	0.16	0.75	0.37	0.99	0.45	1.48	0.11	0.11	1.58	0.13	0.19	0.02	0.16	0.06	0.16	0.05	0.05	0.52
**9**	6.34	1.46	3.82	4.35	13.49	3.57	23.80	14.38	1.65	16.80	7.67	2.19	3.36	2.18	0.66	1.53	0.70	0.30	2.33
	0.30	0.09	0.22	0.06	0.66	0.34	0.84	0.12	0.05	0.84	0.26	0.04	0.08	0.10	0.02	0.11	0.02	0.02	0.24
**10**	7.02	1.62	4.70	4.34	14.94	3.96	17.60	10.46	1.68	15.40	6.87	2.24	1.80	1.25	0.46	0.79	0.63	0.37	1.79
	0.23	0.08	0.20	0.15	0.50	0.16	0.89	0.07	0.08	1.14	0.19	0.10	0.06	0.10	0.05	0.13	0.06	0.06	0.53
**11**	6.80	1.56	4.11	4.36	14.46	3.57	23.60	13.19	1.79	21.40	7.83	2.73	2.68	2.37	0.78	1.59	0.67	0.33	2.05
	0.21	0.05	0.55	0.08	0.45	0.48	2.07	0.05	0.15	1.14	0.28	0.05	0.12	0.19	0.07	0.22	0.04	0.04	0.44
**12**	5.54	1.54	4.16	3.60	12.03	4.34	18.00	13.07	1.38	15.00	7.35	2.04	2.86	1.73	0.54	1.19	0.69	0.31	2.30
	0.38	0.05	0.66	0.20	0.75	0.53	0.71	0.19	0.04	1.58	0.16	0.17	0.04	0.22	0.09	0.22	0.06	0.06	0.69
**13**	3.80	1.06	4.01	3.59	8.26	6.11	18.20	13.24	1.37	13.60	7.85	1.73	2.70	1.50	0.42	1.08	0.72	0.28	2.75
	0.07	0.05	0.29	0.15	0.17	0.38	1.30	0.04	0.09	0.89	0.22	0.07	0.09	0.23	0.09	0.25	0.07	0.07	1.02
**14**	5.70	1.16	2.58	4.92	11.99	2.71	23.60	15.06	1.57	14.40	7.67	1.88	3.70	2.21	0.65	1.56	0.70	0.30	2.40
	0.19	0.05	0.28	0.16	0.40	0.33	1.14	0.24	0.05	1.82	0.08	0.22	0.09	0.37	0.02	0.37	0.06	0.06	0.59
**15**	5.24	1.36	2.67	3.86	11.29	2.97	23.00	15.60	1.48	18.40	6.35	2.89	4.62	3.93	0.55	3.38	0.86	0.14	6.42
	3.41	3.95	0.17	0.19	0.54	0.17	0.00	0.62	0.06	2.79	0.23	0.34	0.21	0.48	0.11	0.51	0.04	0.04	1.95
**16**	4.48	1.00	2.76	4.48	9.50	3.66	21.40	13.42	1.61	16.60	6.99	2.38	3.21	2.29	0.50	1.80	0.78	0.22	3.65
	0.33	0.07	0.57	0.10	0.70	0.78	1.52	1.75	0.13	1.14	0.60	0.13	0.78	0.27	0.05	0.26	0.03	0.03	0.60
**17**	6.24	1.58			13.41	3.20	26.80	16.40	1.63	17.00	7.40	2.30	4.50	1.57	0.52	1.05	0.67	0.33	2.02
	0.52	0.08	0.27	0.18	1.06	0.09	1.30	0.72	0.02	0.71	0.50	0.09	0.16	0.07	0.03	0.07	0.02	0.02	0.19
**18**	5.82	1.10	4.60	5.30	12.18	4.74	20.00	11.43	1.75	20.00	7.38	2.71	2.03	3.66	0.60	3.06	0.84	0.16	5.11
	0.51	0.12	0.55	0.19	1.08	0.35	1.41	0.32	0.10	1.00	0.33	0.09	0.05	0.19	0.04	0.20	0.01	0.01	0.55
**19**	5.36	1.26	3.82	4.28	11.43	4.19	17.80	10.67	1.67	14.80	6.83	2.17	1.92	1.25	0.49	0.76	0.61	0.39	1.57
	0.11	0.11	1.04	0.31	0.32	1.05	1.30	0.12	0.11	0.84	0.19	0.07	0.07	0.08	0.03	0.06	0.01	0.01	0.10

	Matrix File Input																	
Case	1	2	3	4	5	6	7	8	9	10	11	12	13	14	15	16	17	18
2	0.78																	
3	0.74	0.95																
4	0.71	0.91	0.95															
5	0.71	0.91	0.95	1.00														
6	0.74	0.95	1.00	0.95	0.95													
7	0.74	0.95	1.00	0.95	0.95	1.00												
8	0.55	0.71	0.68	0.71	0.71	0.68	0.68											
9	0.68	0.80	0.83	0.88	0.88	0.83	0.83	0.69										
10	0.71	0.91	0.95	0.91	0.91	0.95	0.95	0.66	0.80									
11	0.58	0.69	0.72	0.69	0.69	0.72	0.72	0.55	0.61	0.76								
12	0.56	0.68	0.71	0.68	0.68	0.71	0.71	0.53	0.65	0.68	0.83							
13	0.64	0.76	0.79	0.76	0.76	0.79	0.79	0.55	0.73	0.76	0.69	0.75						
14	0.65	0.77	0.80	0.84	0.84	0.80	0.80	0.61	0.74	0.77	0.64	0.63	0.77					
15	0.60	0.72	0.75	0.79	0.79	0.75	0.75	0.57	0.69	0.72	0.65	0.71	0.79	0.88				
16	0.63	0.75	0.78	0.83	0.83	0.78	0.78	0.59	0.72	0.75	0.68	0.74	0.83	0.83	0.95			
17	0.68	0.73	0.76	0.80	0.80	0.76	0.76	0.58	0.70	0.73	0.67	0.65	0.67	0.81	0.83	0.79		
18	0.72	0.77	0.80	0.84	0.84	0.80	0.80	0.61	0.74	0.77	0.70	0.63	0.77	0.85	0.80	0.76	0.88	
19	0.72	0.77	0.80	0.84	0.84	0.80	0.80	0.61	0.74	0.77	0.70	0.63	0.77	0.85	0.80	0.76	0.88	1.00

Supplementary Figure S1. ISSR-based genetic similarity matrix of historic 'Mehras' olive accessions.

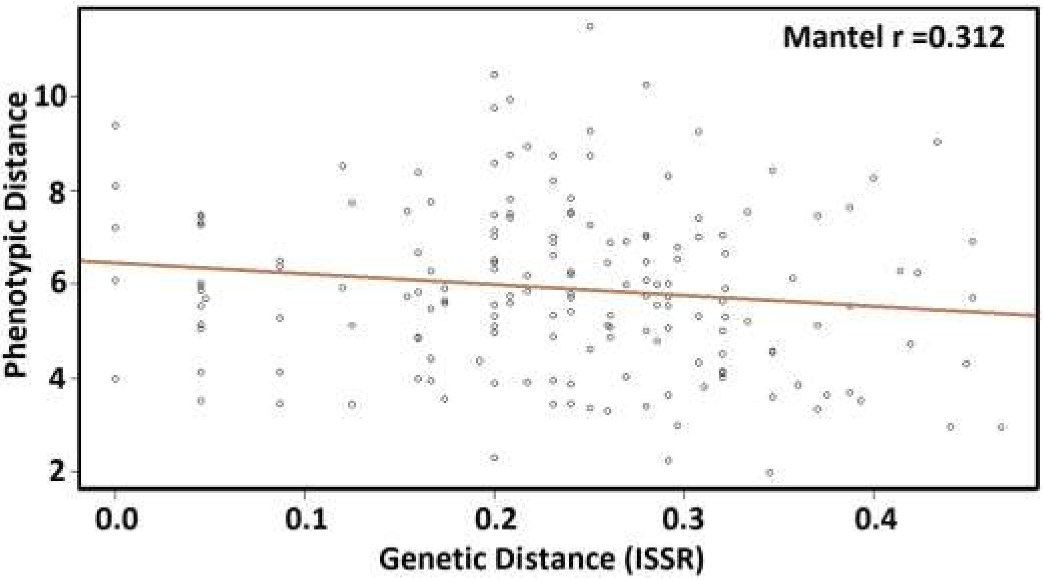

Supplementary Figure S2. Mantel test showing the relationship between genetic and phenotypic distances among 'Mehras' olive accessions.

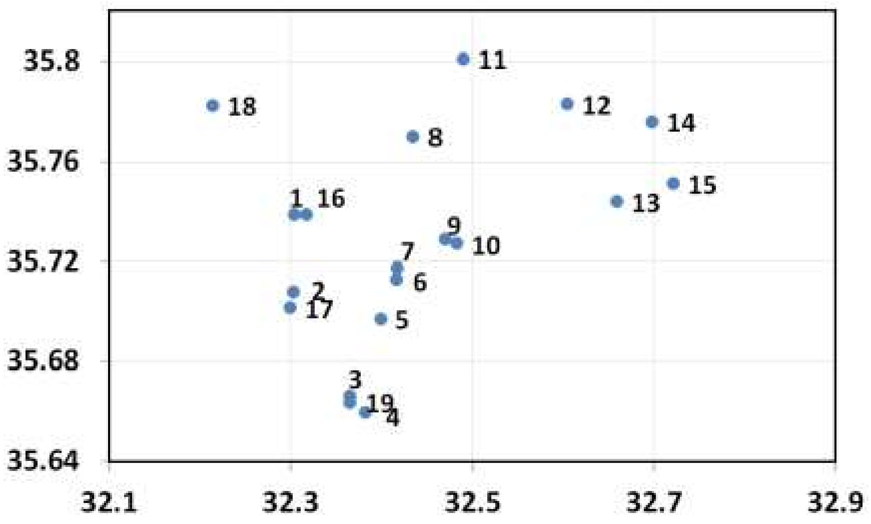

Supplementary Figure S3. Spatial distribution of 'Mehras' olive accessions in relation to genetic and phenotypic patterns across northern Jordan.
